# A qualitative and quantitative analysis of radiation dose and image quality of computed tomography images using adaptive statistical iterative reconstruction

**DOI:** 10.1120/jacmp.v17i3.5903

**Published:** 2016-05-08

**Authors:** Fahad Ahmed Hussain, Noor Mail, Abdulrahman M. Shamy, Suliman Alghamdi, Abdelhamid Saoudi

**Affiliations:** ^1^ Oncology Department King Abdulaziz Medical City Jeddah Mekkah Saudi Arabia; ^2^ King Saud bin Abdulaziz University for Health Sciences Jeddah Mekkah Saudi Arabia; ^3^ King Abdullah International Medical Research Center Jeddah Saudi Arabia

**Keywords:** computerized tomography, ERUS, EUS, multidetector computerized tomography, transrectal, X‐ray

## Abstract

Image quality is a key issue in radiology, particularly in a clinical setting where it is important to achieve accurate diagnoses while minimizing radiation dose. Some computed tomography (CT) manufacturers have introduced algorithms that claim significant dose reduction. In this study, we assessed CT image quality produced by two reconstruction algorithms provided with GE Healthcare's Discovery 690 Elite positron emission tomography (PET) CT scanner. Image quality was measured for images obtained at various doses with both conventional filtered back‐projection (FBP) and adaptive statistical iterative reconstruction (ASIR) algorithms. A standard CT dose index (CTDI) phantom and a pencil ionization chamber were used to measure the CT dose at 120 kVp and an exposure of 260 mAs. Image quality was assessed using two phantoms. CT images of both phantoms were acquired at tube voltage (kV) of 120 with exposures ranging from 25 mAs to 400 mAs. Images were reconstructed using FBP and ASIR ranging from 10% to 100%, then analyzed for noise, low‐contrast detectability, contrast‐to‐noise ratio (CNR), and modulation transfer function (MTF). Noise was 4.6 HU in water phantom images acquired at 260 mAs/FBP 120 kV and 130 mAs/50% ASIR 120 kV. The large objects (frequency<7 lp/cm) retained fairly acceptable image quality at 130 mAs/50% ASIR, compared to 260 mAs/FBP. The application of ASIR for small objects (frequency>7 lp/cm) showed poor visibility compared to FBP at 260 mAs and even worse for images acquired at less than 130 mAs. ASIR blending more than 50% at low dose tends to reduce contrast of small objects (frequency>7 lp/cm). We concluded that dose reduction and ASIR should be applied with close attention if the objects to be detected or diagnosed are small (frequency>7 lp/cm). Further investigations are required to correlate the small objects (frequency>7 lp/cm) to patient anatomy and clinical diagnosis.

PACS number(s): 87.57.‐s, 87.57.C, 87.57.cf, 87.57.cj, 87.57.cm, 87.57.cp, 87.57.N, 87.57.nf, 87.57.np, 87.57.nt, 87.57.Q, 87.59.‐e, 87.59.B

## I. INTRODUCTION

CT is a widespread imaging modality and heavily used in clinics. This modality reveals significant diagnostic information, but it also carries a potential of hazard due to the large dose delivered to patients. Therefore, the medical physics community has been always concerned about the tradeoff between radiation dose and image quality. As per radiation safety rules, increased radiation exposure increases the risk of radiation‐induced cancer. Therefore, we have to acquire the images using minimum radiation possible to minimize the risk. However, reducing the radiation dose carries a risk of compromising image quality due to increased noise. Since CT images were first used in a clinical setting, the standard method of image reconstruction has been filtered back‐projection (FBP).^(1)^ This is simple back‐projection of the X‐ray signal into the image matrix and a filter applied to make the objects sharper. Some CT manufacturers have introduced algorithms that utilize iterative reconstructions and they claim to reduce the patient radiation dose while preserving image quality. The concept of iterative reconstruction has existed since the 1960s, but it was hard to implement clinically due to modest computational power. The power of today's computers has brought iterative reconstruction to the forefront once again.

In addition to FBP, ASIR has been used clinically in our institution since 2011. Since then we noticed some oversmoothing and reduced sharpness on ASIR images. Thereafter, we conducted this study to assess the impact of ASIR on image quality to make sure ASIR did not compromise the diagnostic information of the CT images. ASIR is an iterative reconstruction technique based on the repetitive reconstruction of a set of image data, and each iteration leads to a better representation of the object being imaged. ASIR is based on noise and photon statistics modeling that allows reconstruction to be performed fast enough, within minutes, for clinical applications. The process consists of three main steps. First, artificial raw data are created by forward‐projection of the object estimate. Second, correction terms are generated by comparing the artificial raw data to the measured raw data. The final step involves back‐projection of the correction term onto the volumetric object estimate. The iteration loop stops when the correction to the current estimated image is sufficiently small, a criterion of image quality is met, or a certain number of iterations is reached.

In ASIR, the reconstruction is seeded with initial information (y) obtained from the FBP algorithm, which it then transforms using matrix algebra into a new estimated pixel value $\left(y^{\prime }\right)$. Next, $y^{\prime }$ is compared with an ideal value estimated by the noise model. This process continues iteratively until the estimated value converges on the ideal value.^2^, ^3^ The final pixel value $\hat{A}$ can be expressed as:

(1) $$\hat{A}=\text{arg}\;\text{min}\;\{L(Ax,y)+\alpha G(x)\}$$

where α G(x) is a regularization function and *L* is a cost function.

A previous study of colonography^(4)^ using ASIR and a radiation dose half of the routinely used level concluded that image quality did not deteriorate significantly when compared to that obtained with FBP alone. Vorona et al.^(5)^ reported a 37% dose reduction at 40% ASIR with acceptable image quality for a pediatric abdominal CT. Another study^(6)^ at low X‐ray tube voltage and high X‐ray tube current showed that noise was significantly reduced using low‐dose ASIR and that the CNR was improved for some anatomical organs (the aorta and pancreas) but remained unchanged for other organs (the liver). Chest diagnosis using ASIR and ASIR‐high definition (ASIR‐HD) has shown acceptable image quality or improved confidence.^7^, ^8^, ^9^ Hara et al.^(10)^ reported that using ASIR with a 50% lower radiation dose compared to the routine dose can still fulfill the American College of Radiology (ACR) criteria in terms of low and high contrast;^(11)^ however, the high‐contrast resolution is better when using the standard dose.

To date, most studies in this area have reported the performance of low‐dose ASIR using qualitative measures (i.e., reports of “acceptable image quality,” “insignificant degradation of image quality,” and so on), but have not provided quantitative measures of the performance offered. Other studies have reported oversmoothing and degraded high‐contrast resolution using low‐dose ASIR.^2^, ^10^ To our knowledge, the amount of loss/gain achieved using this approach has not yet been quantified. The aim of this research was to quantify the image information that is gained or lost with low‐dose ASIR, especially in the high spatial‐frequency zone. Image quality parameters, such as noise, contrast, contrast‐to‐noise ratio (CNR), and modulation transfer function (MTF), are presented. Opinions of the radiologists on clinical images are also reported.

## II. MATERIALS AND METHODS

In this study, we used our PET/CT scanner, a Discovery 690 Elite (GE Healthcare, Waukesha, WI), which was equipped with ASIR. A standard CT dose index (CTDI) phantom (body phantom 32 cm thick, head phantom 16 cm thick, both composed of acrylic cylinders 15 cm tall) was used for dose measurements. A pencil ionization chamber attached to an electrometer was used to measure the CT dose on the CTDI phantom (PTW‐Freiburg GmbH, Freiburg, Germany). Noise was measured using a uniform water phantom (model 2144715, rev.8, S/N 1007923), provided by GE for CT daily QC. Image quality was assessed using a Catphan 504 phantom (Phantom Laboratory, Greenwich, NY), which performs a complete, comprehensive evaluation of CT image quality, assessing scan‐slice geometry, image resolution, phantom position, patient alignment, low‐contrast sensitivity, spatial uniformity, scan incrimination and circular symmetry. Images were analyzed using the MATLAB image analysis software (version R2009a, MathWorks Inc., Natick, MA) and direct readout from the CT workstation.

### A. Dose

Dose measurements were performed on a body phantom using adult abdomen protocol (120 kV, 260 mAs, 5 mm slice thickness, scan field of view (SFOV) large body, displayed field of view (DFOV) 50 cm, detector coverage 40 mm, and acquisition 5 mm slice×8i (images per rotation)). The CTDI phantom was placed at the center of the bore aligned with the lasers; the axial, sagittal and coronal lasers coincided with the corresponding phantom markers. The scan was performed in axial mode to measure central and peripheral dose in air kerma using a 100 mm ion chamber that collects liberated electrons (in units of pC). The electrometer calibration factor $(8.71\times 10^{10})\;\text{mGy}.\text{cm}/C$ was used to convert the charges into the units of air kerma (cGy). The final ${\text{CTDI}}_{w}$ was calculated by weighting the peripheral and central dose and then correcting measured air kerma for room pressure and temperature. We measured the dose at 260 mAs, and then used the linear relation between dose and mAs to estimate the dose for the rest of the mAs settings. Most of the image quality comparisons were done at 260 mAs/FBP 120 kV and 130 mAs/50% ASIR 120 kV, because these protocols are routinely used in the clinic in our institution. The image quality as a function of % ASIR was studied to evaluate image quality response to different levels of ASIR.

### B. Image noise as a function of ASIR level

Hereafter, the standard deviation (SD) of CT number is referred to as image noise. For accurate noise measurement, we used water phantom for three reasons. First reason was the high uniformity of water; second was the usage of water CT number as a standard reference CT number calculation for other materials; and, last, water consists ∼70% of the human body. The water section of the GE QC phantom was scanned over a range of different acquisition parameters and noise measurements were taken. Images were acquired using a single tube voltage (120 kV) and two tube currents (260 and 130 mAs). Two reconstruction methods were used: FBP and ASIR (10%–100% in 10% increments). All scans were acquired using one single axial rotation. A region of interest (ROI) was selected covering 80% of the water phantom at the center (approximately equal to 22,000 mm^2^) at 5 mm slice thickness. Noise (SD) was recorded for each image and reconstruction technique.

Percentage ASIR (% ASIR) means the strength of blending between FBP and image that resulted from reconstruction iterations. For example, ASIR 40% is a reconstruction blending of 40% ASIR and 60% FBP.^12^, ^13^, ^14^

### C. MTF as a function of ASIR level

The Catphan 504 was aligned in the middle of the CT scan field of view using the alignment lasers. The positioning section was scanned to ensure accurate alignment of the phantom with the CT image plane. All four alignment markers (BBs) were clearly visible and equally bright at the alignment slice. Next, the high‐contrast‐resolution (HCR) section was scanned, one axial scan, at two different settings; 260 mAs/FBP 120 kV and 130 mAs/50% ASIR 120 kV. Further scans of the HCR bars were obtained at 120 kV and exposures of 25 mAs, 130 mAs, 260 mAs, and 400 mAs to assess the impact of ASIR on MTF at different doses.

MTF was calculated using a practical method based on SD measurements of high‐contrast resolution line pairs, as outlined in Droege and Morin.^(15)^ The MTF is basically the visibility of any object on an image. The high contrast resolution bars show better visibility when SD is high, while visibility degrades as the SD reduces. Therefore, SD can be correlated to MTF. The MTF is calculated using the following formula:

(2) $$MTF(f)=\frac{\pi \sqrt{2}SD(f)}{4SD_{0}}$$

where $SD_{0}$ is the standard deviation at which we consider the bars show 100% contrast (i.e., MTF=1). It is calculated for equal sized region of interest (ROI) by measuring the CT number of the bar material (${\text{CT}}_{m}$) and CT number of the background material (${\text{CT}}_{b}$) using the following formula:

(3) $$SD_{0}=\frac{\left|CT_{b}-CT_{m}\right|}{2}$$

where *SD(f)* is the standard deviation of the resolution bars and was calculated using the following formula:

(4) $$SD(f)=\sqrt{M^{2}-N^{2}}$$

where *M* is the standard deviation of the ROI placed on the resolution bars, and *N* is the background noise, calculated as follows:

(5) $$N^{2}=\frac{N_{b}^{2}+N_{m}^{2}}{2}$$

Here $N_{b}$ is the background noise of the phantom material and $N_{m}$ is the noise of the resolution bar material.

### D. Low‐contrast resolution as a function of ASIR level

The low‐contrast‐resolution section was scanned at 120 kV/260 mAs and 120 kV/130 mAs. These images were reconstructed with slice resolution of 5 mm using FBP and %ASIR levels. Low‐contrast resolution was measured by calculating the CNR for each image of the 15‐mm‐diameter supraslice object. Supraslice objects are three groups of nine contrast objects varying in diameter from 2‐15 mm and each group has a contrast of 1%, 0.5%, and 0.3%, respectively. A circular ROI was drawn that covered 80% of the area of the largest (15‐mm diameter) low‐contrast object in each supraslice group. The mean and standard deviation of the CT number were measured. Then the same ROI was placed outside the contrast object to measure the mean and noise of the CT number for the background. The location of the ROI was the same for all slices and readings were taken directly from the CT workstation. The two noises were averaged in and out of the contrast object. CNR was calculated using the following formula:^(16)^

(6) $$\begin{array}{l} CNR=\frac{OCT-BCT}{N} \end{array}$$

where *OCT* is object CT number, *BCT* is background CT number, and *N* is the average noise. We compared two sets of images (260 mAs/FBP vs. 130 mAs/50% ASIR), as these two reconstruction techniques resulted the same noise in images of the water phantom. So we checked for the CNR whether it retained the same CNR value or differed.

### E. Clinical case with ASIR application

The clinical study shown here is just an example for high‐frequency objects where ASIR application degrades visibility compared to FBP images. In clinical perspective, ASIR may need further investigation on a large size of sample or a greater number of patients. We conducted this study on a patient after getting his consent form and having the case approved by our institutional review board (IRB). A precontrast scan was acquired using 130 mAs and then a postcontrast was acquired using 260 mAs. Both scans were acquired in the same CT examination and we made sure the patient did not move between two scans. Both set of scans were reconstructed using ASIR from 10% to 100%. The evaluations on the images were carried out on an anatomic area that was unaffected by the injected contrast and respiratory motion.

## III. RESULTS AND DISCUSSION

### A. Dose

For the abdomen protocol (120 kV, 260 mAs, 5‐mm slice, body phantom), we measured the dose at the center and periphery of the phantom. The ${\text{CTDI}}_{w}$ was then calculated as 21.7 mGy, which was within the tolerance values permitted by the ACR (35 mGy for an adult abdomen). Utilizing the linear relation between radiation output and X‐ray tube current, we estimated the phantom dose at 25 mAs, 130 mAs, and 400 mAs, and then converted them to ${\text{CTDI}}_{w}$ to be 2.1 mGy, 10.8 mGy, and 33.4 mGy, respectively. All of them were within the tolerance of ACR.

### B. Image noise as a function of ASIR level

**Figure 1:**
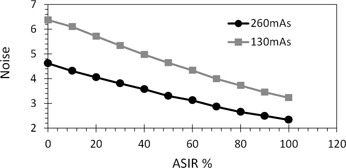
Image noise over 80% of the area of the GE QC water phantom. ASIR varying from 10%–100% and we see noise falls as ASIR % increase for two doses. Image acquired at 130 mAs/50%ASIR achieved almost same noise, 4.6, of that image acquired at 260 mAs/FBP.

Image noise steadily reduced as ASIR level increased from 10% to 100% (Fig. 1). The noise was found to be 4.62 when the image was reconstructed using standard FBP, but fell to 3.3 HU with image reconstruction using 50% ASIR and further decreased to 2.3 HU with image reconstruction using 100% ASIR. When the dose was halved (from 260 to 130 mAs), the noise increased to 6.37 HU when applying FBP. However, it was found to be 4.65 HU with 50% ASIR and 3.2 HU with 100% ASIR. Comparison of these results revealed that the image acquired with 50% lower dose (130 mAs) and 50% ASIR provided almost the same noise like the image acquired with full dose (260 mAs) using standard FBP.

### C. MTF as a function of ASIR level

At a fixed image acquisition parameters (kV, mAs) and voxel size, MTF of large objects improved with the application of ASIR, compared to that obtained with FBP. However, MTF degraded with application of ASIR for small objects. The spatial frequency at which the transition occurred, from improved to degraded MTF, was proportional to the amount of exposure applied. The higher the exposure, the higher the spatial frequency at which ASIR yielded higher MTF than FBP. The transition points at 100% ASIR were 5.7, 6.7, 7, and 7.8 at 25, 130, 260, and 400 mAs respectively (2, 5). So, the ASIRs capability of improving object visibility and differentiating between useful information and noise was depended on the mAs.

The spatial frequency of 50% MTF was always increasing with % ASIR regardless of dose because it is in the range of low‐spatial‐frequency objects. In contrast, the spatial frequency of 10% MTF showed a clear dependence on dose and the level of ASIR applied (6, 7). The frequency of 10% MTF increased consistently at doses 260 and 400 mAs, but almost did not change at 130 mAs. However, spatial frequency of 10% MTF degraded with % ASIR at a dose of 25 mAs. It seemed like the ASIR algorithm started considering the resolution bars, which were visible at 260 and 400 mAs, as noise that needed to be smoothed.

**Figure 2:**
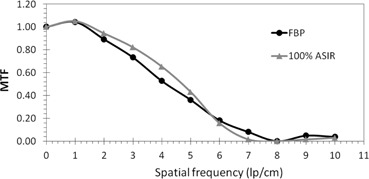
MTF FBP vs. 100% ASIR at a dose of 25 mAs. The spatial frequency at which ASIR yields lower MTF than FBP is approximately 5.7 lp/cm.

**Figure 3:**
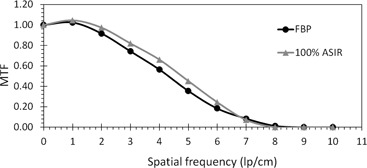
MTF FBP vs. 100% ASIR at a dose of 130 mAs. The spatial frequency at which ASIR yields lower MTF than FBP is approximately 6.7 lp/cm.

**Figure 4:**
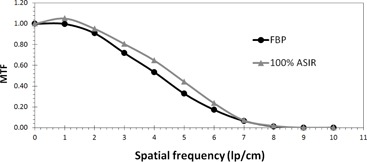
MTF FBP vs. 100% ASIR at a dose of 260 mAs. The spatial frequency at which ASIR yields lower MTF than FBP is approximately 7 lp/cm.

**Figure 5:**
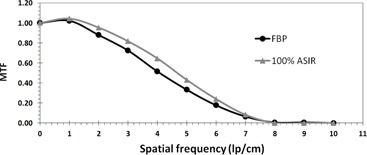
MTF FBP vs. 100% ASIR at a dose of 400 mAs. The spatial frequency at which ASIR yields lower MTF than FBP is approximately 7.8 lp/cm.

**Figure 6:**
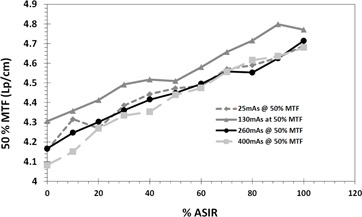
The change of 50% MTF as function of % ASIR at different doses showing increasing 50% MTF with ASIR for all doses and all % ASIR levels.

In Fig. 8, the image acquired with 50% lower dose (130 mAs) and reconstructed using 50% ASIR had a better MTF (i.e., 0.399 at 5 lp/cm) than the image acquired with full dose (260 mAs) using standard FBP (0.328 at 5 lp/cm). This improved MTF held true for spatial frequencies smaller than 7.4 lp/cm. However, objects of frequencies higher than 7.4 lp/cm (at 130 mAs/50% ASIR) exhibited degradation compared to those reconstructed with 260 mAs/FBP. Perhaps it was due to noise definition (i.e., the CT number variation was considered by the algorithm to be noise). The algorithm could not differentiate between the resolution bars and image noise. Therefore the algorithm started averaging the signal whereby ASIR resulted in lower MTF for frequency >7.4 lp/cm. Although image noise was the same, for both sets of image acquisition parameters, in water, the image quality with Catphan phantom differed when measured in terms of MTF.

**Figure 7:**
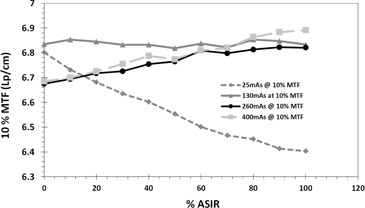
The change of 10% MTF as function of % ASIR at different doses showing dose dependence and % ASIR dependence.

**Figure 8:**
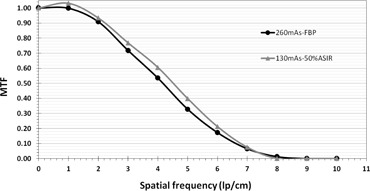
MTF as a function of spatial frequency for images reconstructed with standard FBP and a full radiation dose of 260 mAs (circles) and 50% ASIR with half of the radiation dose of 130 mAs (triangles). Above a frequency of 6.7 lp/cm, the ASIR technique with lower dose does not offer a better MTF than the standard FBP technique with full dose.

### D. Low contrast resolution (LCR) as a function of ASIR level.

**Figure 9:**
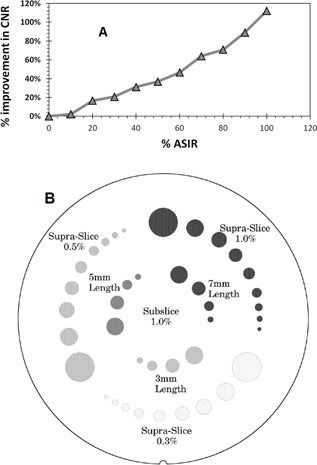
(a) Average CNR of six measurements on all 1%, 0.5%, and 0.3% contrast‐level objects for two doses 130 mAs and 260 mAs. The CNR obtained with ASIR was compared to CNR obtained with FBP. (b) A cross‐sectional illustration of the low‐resolution objects in Catphan.

**Figure 10:**
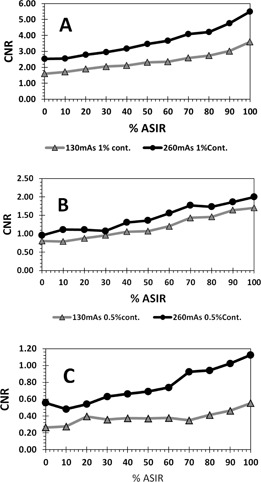
CNR values obtained with ASIR at different levels as a function of dose and for different contrast‐level objects: (a) 1% contrast‐level objects; (b) 0.5% contrast‐level objects; (c) 0.3% contrast‐level objects. In general, there is increase in CNR with higher levels of ASIR. However, for 0.3 contrast objects the CNR change is not significant.

The average CNR improvement at each ASIR level is shown in Fig. 9(a). Each point in the figure represents an average of six CNRs at the same ASIR level. The six CNRs consist 1%‐, 0.5%‐, and 0.3%‐contrast objects, each acquired at 130 mAs and 260 mAs (Fig. 9(b)). The average CNR readily increased with increasing % ASIR. Also, the discrete measurements of CNR for each contrast level objects, showed increased CNR with higher levels of ASIR, as seen in Figs. 10(a) to 10(c). This could be attributed to the decrease in noise with ASIR. However, this trend was not consistent at low dose (130 mAs) for the 0.3%‐contrast‐level objects. We can see in Fig. 10(c) that there was almost no change in CNR from 20% to 70% ASIR. So in this case, CNR did not improve with ASIR, as the 0.3%‐contrast‐level objects disappeared in the additional noise at low dose. The information that disappeared with noise might be significant clinical information. Further investigation is needed into the clinical significance and implications of the information that was lost at low dose with ASIR. Both scans (half‐dose 50% ASIR and full‐dose FBP) produced the same amount of noise, but not the same CNR, as shown in Fig. 10. CNR was always higher for the 260 mAs compared to 130 mAs throughout all % ASIR levels. For 1%‐contrast objects, both images had same CNR at 65% ASIR, while the 0.5%‐contrast objects had same CNR at 30% ASIR. The 0.3%‐contrast objects had the same CNR at 100% ASIR.

### E. Clinical case with ASIR application

**Figure 11:**
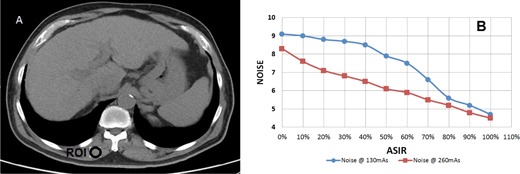
(a) A cross‐sectional area of the patient. Noise was measured in the ROI appearing on the image. The ROI was selected on a location that is not affected by respiratory motion and contract. (b) The noise of ROI 1 as a function of ASIR level. 0% ASIR refers to image reconstruction using standard FBP. We notice a consistent reduction in noise with ASIR. The 130 mAs image meets the same noise of 260 mAs at around 45% ASIR.

The ROI indicated in Fig. 11(a) was the area we used to measure the noise on the patient image. This area was unaffected by respiratory motion and contrast. The noise in the ROI consistently reduced with ASIR (Fig. 11(b)). At approximately 45% ASIR, the 130 mAs resulted same noise as 260 mAs/FBP. So this was a very close match to the phantom study results; same noise at 260 mAs/STD/FBP vs. 130 mAs/50% ASIR.

**Figure 12:**
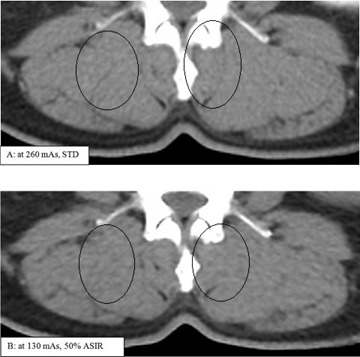
Patient images taken using different image reconstruction algorithms and doses. (a) Using standard FBP at a dose of 260 mAs; (b) using 50% ASIR at a dose of 130 mAs. (a) is showing more fine details than (b), although both settings produced same noise in water phantom. We can see some fatty plaques (in (a)) have disappeared in (b); also, some other fatty plaques that did not appear in (a) have been exaggerated in (b).

Having two combinations of image reconstruction techniques producing equal noise, raised the question of image quality at same conditions. However, as we can see from Fig. 12, the ASIR image taken at half‐dose had reduced sharpness and contrast. We could see that some fatty plaques in the paravertebral muscle in Fig. 12(a) have disappeared in (b), and also that some other fatty plaques that did not appear in (a) have been exaggerated in (b). This was perhaps due to the oversmoothing applied by ASIR to the pixels of close values.

Generally, we noticed that ASIR reconstructed acceptable images with blending strength less than 50%. ASIR blending strength more than 50% resulted in vague images. However, noise consistently reduced with ASIR throughout all levels of ASIR, resulting in better CNR for uniform anatomy; but it caused loss of sharpness affecting the image quality significantly, especially for high frequency objects.

**Figure 13:**
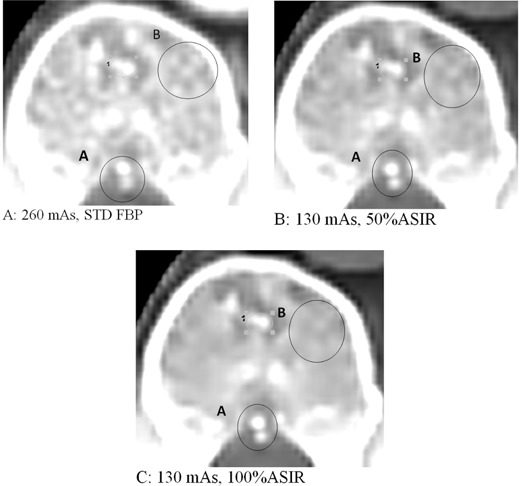
Image of vertebrae showing the change in image quality at (a) 260 mAs/FBP vs. (b) 130 mAs/50% ASIR and (c) 130 mAs/100% ASIR. The visibility of ROI‐A improved but, for ROI‐B, it degraded.

Figure 13 shows an image of vertebrae taken with two different ASIR levels and FBP for comparison. We clearly noticed that ASIR enhanced image quality in some areas like ROI‐A, but degraded image quality in other areas like ROI‐B, depending on the size and contrast of anatomy. This clinical study matched the phantom study, where we saw ASIR yielded better or degraded MTF depending on the size of the object. This threshold size and contrast depends on the scanning dose delivered to patient, as shown in 2, 5.

**Figure 14:**
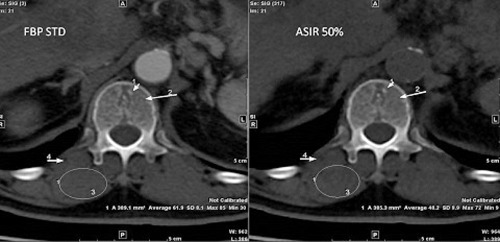
Patient images acquired at 260 mAs and reconstructed with standard FBP (left) and 130 mAs 50% ASIR (right). We can clearly see change in image quality although phantom study showed that both image acquisition settings produce same noise.

**Figure 15:**
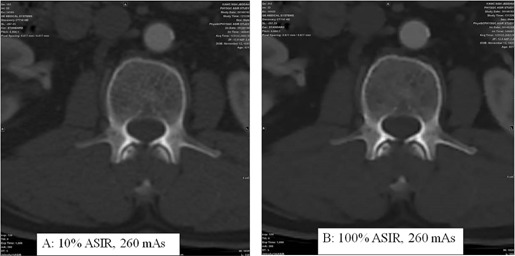
Abdominal images acquired at 120 kVp and 260 mAs. Trabecular bony elements with 10% ASIR (a) are much better visualized than the 100% ASIR (b).

In Fig. 14, trabecular bony elements were better defined at 260 mAs without ASIR, as indicated by arrow 1 and arrow 2. Trabecular bony elements information loss occurred with 50% ASIR at 130 mAs (Fig. 14, right panel). Similarly, the right paravertebral muscle (indicated by the superimposed circles in Fig. 14) was poorly defined with ASIR compared to FBP images. Fatty plaques (indicated by arrow 4) were poorly defined with 50% ASIR compared to 260 mAs FBP. The vertebral hemangioma, which is the most common benign vertebral neoplasm, was diagnosed with typical intralesional bony trabecular, the polka dot sign. The trabecular bony elements, right paravertebral muscle, and vertebral hemangioma could be seen more clearly in Fig. 15 with 10% ASIR than with 100% ASIR. These findings on patients consolidated the findings of the phantom study: ASIR improved quality of some parts of the image but degraded other parts according to the type and size of the anatomy. Patient study showed that having the same image noise did not mean having the same image quality.

## V. CONCLUSIONS

We have studied the effect of ASIR on phantom image quality and verified the findings on patient images. ASIR offers improvement in image quality in terms of CNR and MTF, depending upon the level of radiation exposure. At low doses, ASIR cannot distinguish between a low‐contrast object (for example, a 0.3% contrast‐level object) and noise. The capability of ASIR to improve MTF works up to a certain threshold spatial frequency; beyond a certain high frequency, the use of ASIR actually degrades MTF. This threshold changes to higher or lower values depending on the scanning dose used to obtain the image. When MTF or sharpness starts degrading, that means some information, that might be clinically important, is removed from the image. Therefore, physicians need to be careful when acquiring CT images using ASIR. To diagnose very small objects, (frequency>6 lp/cm), we think it would be better to use standard dose with FBP or a low level of ASIR (<30% ASIR). Further study can be carried out to identify the exact anatomical information, for different organs or examinations, that is lost when sharpness and MTF start degrading with ASIR, and to identify the importance of that in patient diagnosis accuracy. So in this study we identified the threshold frequency whereby ASIR starts degrading MTF and we showed the effect of this on trabecular bony elements.

## ACKNOWLEDGMENTS

We would like to thank our oncology and radiology departments in our institution for the support in this study.

## COPYRIGHT

This work is licensed under a Creative Commons Attribution 4.0 International License.

## Supporting information

Supplementary Material

Supplementary Material
