# Supplementary figures and images for: A qualitative and quantitative analysis of radiation dose and image quality of computed tomography images using adaptive statistical iterative reconstruction
Source: J Appl Clin Med Phys. 2016 May 8;17(3):419–32. doi: 10.1120/jacmp.v17i3.5903 (PMC5690909; doi:10.1120/jacmp.v17i3.5903)

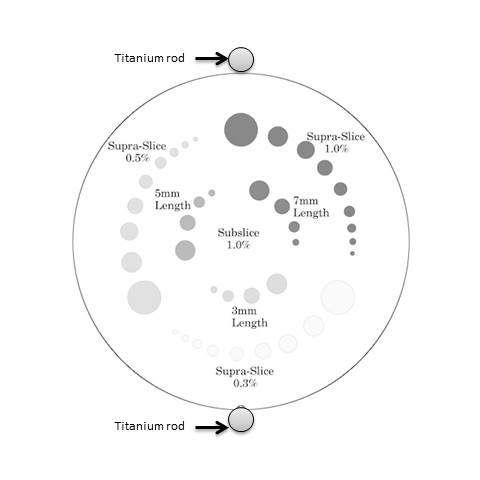

Supplement: Supplementary file 1 — Supplementary Material [file ACM2-17-419-s001.jpg]
