# Supplementary material for: A qualitative and quantitative analysis of radiation dose and image quality of computed tomography images using adaptive statistical iterative reconstruction
Source: J Appl Clin Med Phys. 2016 May 8;17(3):419–32. doi: 10.1120/jacmp.v17i3.5903 (PMC5690909; doi:10.1120/jacmp.v17i3.5903)
Supplement: Supplementary file 2 — Supplementary Material [file ACM2-17-419-s002.pdf]

June 16, 2015

Editor-in-Chief:
Michael D. Mills, Ph.D.
Brown Cancer Center,
Department of Radiation Oncology,
The University of Louisville
529 South Jackson Street
Louisville, KY 40202 USA

Dear Dr. Michael D. Mills,

We are submitting a manuscript entitled: "**A qualitative and quantitative analysis of radiation dose and image quality of computed tomography images using Adaptive Statistical Iterative Reconstruction**". In this study, we assessed (CT) image quality produced by two reconstruction algorithms provided with GE Healthcare's Discovery 690 Elite positron emission tomography (PET)/CT scanner. Image quality was measured for images obtained using varying doses of radiation with both conventional filtered back projection (FBP) and adaptive statistical iterative reconstruction (ASIR) algorithms. ASIR has been clinically implemented here in our health care institute since 2013, therefore this work has importance for those institutions which uses ASIR in the clinic.

Please do not hesitate to contact me by e-mail or phone if you have any questions or if there is anything else that I can provide. Thank you very much for your consideration.

Sincerely,

NOOR MAIL, MS, MSc, MPhil, PhD, DABR
Senior Medical Physicist and Head of Research, Radiation Oncology
Princess Noorah Radiation Oncology Center
Ministry of National Guard Health Affairs, Jeddah,
P.O.Box 9515, Jeddah 21423, Kingdom of Saudi Arabia
Tel: 00966-012-2266666 Ext. 24074-6
